# Optimising nutrition during therapeutic hypothermia

**DOI:** 10.1136/archdischild-2018-315393

**Published:** 2018-10-15

**Authors:** Shalini Ojha, Jon Dorling, Cheryl Battersby, Nicholas Longford, Chris Gale

**Affiliations:** 1 Division of Medical Sciences and Graduate Entry Medicine, School of Medicine, University of Nottingham, Nottingham, UK; 2 Division of Neonatal-Perinatal Medicine, Faculty of Medicine, IWK Health Centre, Dalhousie University, Halifax, Nova Scotia, Canada; 3 Section of Neonatal Medicine, Department of Medicine, Imperial College London, London, UK; 4 Neonatal Data Analysis Unit, Section of Neonatal Medicine, Department of Medicine, Imperial College London, London, UK

**Keywords:** infant feeding, parenteral nutrition, hypothermia, induced, hypoxia-ischaemia, brain, NNRD, infant, newborn, intensive care, neonatal

There is little evidence to inform provision of enteral or parenteral nutrition to infants with hypoxic ischaemic encephalopathy (HIE) during and soon after therapeutic hypothermia; as a consequence, clinical practice is both variable and changing. A 2014 UK survey found that 79% (33 of 42) of responding neonatal units routinely withheld enteral nutrition during cooling; 3 years later, a similar survey found that 41% (20 of 49) of responding units reported withholding enteral nutrition.^1^ The latter study also reports wide variation in how, when and how much to feed, and in the use of parenteral nutrition. Internationally, practice is even more variable: withholding enteral feeds is practised almost universally^2^ in some countries, while in others, milk feeding during hypothermia is routine.^3^ Here we discuss the limited evidence available to inform enteral and parenteral nutrition during therapeutic hypothermia.

## Enteral nutrition

The landmark trials^4^ that provided evidence for the neurodevelopmental benefits of therapeutic hypothermia withheld milk feeds during hypothermia. It is therefore unsurprising that withholding or delaying enteral feeding is commonplace. A major rationale for this practice is to prevent necrotising enterocolitis (NEC). NEC is well described in term infants with HIE, but is rare: among 11 trials, with a total of 1505 infants, included in the Cochrane review of therapeutic hypothermia only three trials reported data on NEC and only one case was identified (in the hypothermia arm of the TOBY (Whole Body Hypothermia for the Treatment of Perinatal Asphyxial Encephalopathy) trial.^4^ Although the pathogenesis of NEC is incompletely understood, one putative mechanism links the combination of impaired gastrointestinal blood flow (as seen in infants with HIE) and intraluminal substrate (such as milk) to the proinflammatory cascade that leads to gut necrosis and NEC. As a consequence, ‘prevention of NEC’ is a common reason for withholding enteral feeds in infants receiving therapeutic hypothermia for HIE, despite lack of evidence that withholding enteral feeds in other high-risk groups (such as preterm infants) reduces the incidence of NEC.^5^ ^6^

The gut plays a crucial role in the pathophysiology of critical illnesses such as HIE and NEC. The epithelial lining of the gut acts as a barrier, and plays an important role in preventing translocation of bacteria and other damaging luminal contents into the systemic circulation; inflammatory mediators released by the enteric immune system have local intestinal and systemic effects. In this context, enteral nutrition, particularly with mother’s own milk, may actually play a beneficial role by influencing structural and functional integrity of the gut, reducing systemic inflammatory responses and promoting proliferation of gut microbial diversity. The limited available clinical data support this: a retrospective matched case–control study of 34 infants in the USA, which compared minimal enteral nutrition with withholding feeds during therapeutic hypothermia, found lower inflammatory cytokine concentrations and a reduction in days of parenteral nutrition and hospital stay in the enteral feeding group.^2^ Similarly, a retrospective cohort study of 85 infants that compared outcomes in the UK and Sweden where 33% vs 91% of infants received milk feeds during hypothermia respectively, found no differences in clinical outcomes (and no cases of NEC in either group).^3^ Animal model data suggest that hypothermia may have a protective effect on the gut by attenuating the systemic oxidative stress response and reducing inflammation and metabolic injury.^7^ The sparse available clinical data support this supposition: a retrospective cohort study including 36 infants who received therapeutic hypothermia for HIE, and 32 historical controls who did not, found that infants in the therapeutic hypothermia cohort reached full milk feeds sooner; furthermore, three cases of NEC were reported—all in the historical (uncooled) cohort.^8^ In summary, the limited evidence suggests that following a hypoxic insult, both hypothermia and enteral feeding may have a protective effect on the gastrointestinal system and careful introduction of enteral feeds appears to be well tolerated and safe.

## Parenteral nutrition

In high-income settings, infants who receive therapeutic hypothermia receive some form of intravenous fluid support: commonly intravenous dextrose with electrolytes as required, or parenteral nutrition.^1^ This practice is also variable—Hazeldine *et al* found that 29% (14/49) of responding UK neonatal units report routine use of parenteral nutrition.^1^ While dextrose provides sufficient carbohydrate energy to prevent hypoglycaemia, it does not limit catabolism as effectively as parenteral nutrition. This is the rationale for giving parenteral nutrition to infants receiving therapeutic hypothermia: to improve overall growth, brain growth and potentially neurodevelopment—although we note that this conjecture is not backed up by any human studies.

In critically ill adults and children the provision of early parenteral nutrition is increasingly controversial, with accumulating evidence of harm caused by early parenteral nutrition. A systematic review of 18 adult randomised controlled trials, with a total of 3347 patients, found no effect on mortality but significantly higher rates of infection and longer average length of stay in patients in the parenteral compared with enteral nutrition groups.^9^ The Early versus Late Parenteral Nutrition in the Pediatric Intensive Care Unit (PEPaNIC) trial reached a similar conclusion, that early provision of parenteral nutrition may be harmful. This trial randomised 1440 critically ill children (including 209 term neonates) to receive parenteral nutrition within 24 hours of admission, or for parenteral nutrition to be delayed for 7 days. While mortality rates were similar in the two groups, children who had parenteral nutrition withheld for 7 days had a significantly lower rate of new infection (10.7% vs 18.5%).^5^ A preplanned secondary analysis of the 209 term neonates included in the PEPaNIC trial confirmed lower likelihood of infection and higher rate of survival to discharge in infants for whom parenteral nutrition was withheld, but at the cost of an increased risk of hypoglycaemia.^10^ While these results are not directly applicable to term infants undergoing therapeutic hypothermia for HIE, for example, the majority of neonatal participants in the PEPaNIC trial were surgical patients, it does raise concerns about the potential harms of early parenteral nutrition in term infants that require critical care generally.

The evidence to guide nutritional practice for term infants undergoing therapeutic hypothermia is based on a few small studies, with inherent risk of bias. This limited available evidence suggests that careful introduction of enteral feeds during therapeutic hypothermia is safe, may beneficially modulate inflammatory responses and may be associated with earlier time to full enteral feeding and earlier discharge. Parenteral nutrition during critical illnesses in children and term neonates may do more harm by increasing the risk of serious infection and prolonging hospital stay—but the degree to which this is applicable to infants with HIE undergoing therapeutic hypothermia is not known.

Further research is needed to inform nutritional practice for this sizeable group of infants; an ongoing National Institute for Health Research funded study (https://doi.org/10.1186/ISRCTN47404296, registered pre-results) aims to address this uncertainty using UK population data held on the National Neonatal Research Database. Data from several thousand infants who received therapeutic hypothermia during a 10-year period will be included. This study will form two groups that are matched on an extensive list of background variables using propensity scores. The intention of this approach is to reduce the risk of bias from confounders, facilitating the use of observational data to address the question ‘what is the optimum enteral and parenteral nutrition strategy for newborns with HIE during therapeutic hypothermia?’ While we remain unsure of how to deliver nutrition to infants with HIE, it is imperative to consider that enteral feeding could be a beneficial adjunct to therapeutic hypothermia while parenteral nutrition, although a reliable vehicle for nutrient delivery, may increase the risk of infection.

## References

[1] Hazeldine B, Thyagarajan B, Grant M, et al Survey of nutritional practices during therapeutic hypothermia for hypoxic-ischaemic encephalopathy. BMJ Paediatr Open 2017;1:e000022 10.1136/bmjpo-2017-000022 PMC5842999 29637095

[2] Chang LL, Wynn JL, Pacella MJ, et al Enteral feeding as an adjunct to hypothermia in neonates with hypoxic-ischemic encephalopathy. Neonatology 2018;113:347–52. 10.1159/000487848 29510382

[3] Thyagarajan B, Tillqvist E, Baral V, et al Minimal enteral nutrition during neonatal hypothermia treatment for perinatal hypoxic-ischaemic encephalopathy is safe and feasible. Acta Paediatr 2015;104:146–51. 10.1111/apa.12838 25348803

[4] Jacobs SE, Berg M, Hunt R, et al Cooling for newborns with hypoxic ischaemic encephalopathy. Cochrane Database Syst Rev 2013:CD003311 10.1002/14651858.CD003311.pub3 23440789 PMC7003568

[5] Fivez T, Kerklaan D, Mesotten D, et al Early versus late parenteral nutrition in critically ill children. N Engl J Med 2016;374:1111–22. 10.1056/NEJMoa1514762 26975590

[6] Morgan J, Young L, McGuire W Delayed introduction of progressive enteral feeds to prevent necrotising enterocolitis in very low birth weight infants. Cochrane Database Syst Rev 2014(12). 10.1002/14651858.CD001970.pub5 PMC7063979 25436902

[7] Stefanutti G, Pierro A, Vinardi S, et al Moderate hypothermia protects against systemic oxidative stress in a rat model of intestinal ischemia and reperfusion injury. Shock 2005;24:159–64. 10.1097/01.shk.0000168871.60531.6f 16044087

[8] Thornton KM, Dai H, Septer S, et al Effects of whole body therapeutic hypothermia on gastrointestinal morbidity and feeding tolerance in infants with hypoxic ischemic encephalopathy. Int J Pediatr 2014;2014:1–7. 10.1155/2014/643689 PMC4158474 25214853

[9] Elke G, van Zanten AR, Lemieux M, et al Enteral versus parenteral nutrition in critically ill patients: an updated systematic review and meta-analysis of randomized controlled trials. Crit Care 2016;20:117 10.1186/s13054-016-1298-1 27129307 PMC4851818

[10] van Puffelen E, Vanhorebeek I, Joosten KFM, et al Early versus late parenteral nutrition in critically ill, term neonates: a preplanned secondary subgroup analysis of the PEPaNIC multicentre, randomised controlled trial. Lancet Child Adolesc Health 2018;2:505–15. 10.1016/S2352-4642(18)30131-7 30169323

